# Predicting admission to long‐term care and mortality among community‐based, dependent older people in Ireland

**DOI:** 10.1002/gps.5101

**Published:** 2019-04-17

**Authors:** Niamh Aspell, Maria O'Sullivan, Eamon O'Shea, Kate Irving, Chloe Duffy, Rebecca Gorman, Austin Warters

**Affiliations:** ^1^ North Dublin Homecare Ltd. Dublin Ireland; ^2^ Services for Older People, Health Service Executive Community Healthcare Organisation, Ballymun Healthcare Facility Dublin Ireland; ^3^ Trinity College Dubin, Centre for Health Sciences St. James' Hospital Dublin Ireland; ^4^ Centre for Economic and Social Research on Dementia National University of Ireland Galway Ireland; ^5^ School of Nursing and Human Sciences Dublin City University Dublin Ireland

**Keywords:** ageing, balance of care, dementia, domiciliary care, long‐term care

## Abstract

**Objective:**

To identify factors that predict admission to long‐term care (LTC) and mortality among community‐based, dependent older people in Ireland, who were in receipt of formal home support.

**Methods:**

An audit was conducted of all community‐dwelling older adults receiving government funded home support during 2017 in the Dublin North Central, Health Service Executive administrative area. Data were extracted from the Common Summary Assessment Report (CSAR), a mandatory form used in the provision of home support. Multiple logistic regression analysis was used to examine the factors associated with admission to LTC and mortality, with the results presented as odds ratios (OR) and 95% confidence intervals.

**Results:**

The audit comprised 1597 community‐dwelling older adults with a mean age of 83.3 (SD: 7.2) years. The prevalence of transition to LTC and mortality was 8% and 9%, respectively, during the 12‐month period. Factors significantly associated with admission to LTC were “cognitive dysfunction” [OR 2.10 (1.41‐3.14), *P* < .001] and the intensity of home support [OR 1.05 (1.01‐1.06), *P* < .003], as measured by weekly formal care hours. Physical dependency and advanced age (aged 95 years +) were significantly associated with mortality in this population (*P* < .001).

**Conclusion:**

“Cognitive dysfunction” and intensity of formal home support were associated with transition to LTC, while physical dependency and advanced age were associated with mortality. Investment in personalised, cognitive‐specific, services and supports are necessary to keep people with dementia and related cognitive impairments living at home for longer.

**Key points**

- “Cognitive dysfunction” and intensity of formal home support were significantly associated with admittance to long‐term care in community‐dwelling older people.
- Physical dependence and advanced age (95 years +) were significantly associated with mortality, but not of long‐term care.
- The findings support the case for personalised home support models, particularly for older adults with dementia and cognitive impairment, to enable people to live well at home for longer.

## 1 INTRODUCTION

Around the world, populations are rapidly ageing^1^; while many will experience their later life in good health, there are others for whom older age will be characterised by physical and cognitive decline and dependent on others for care and support. Care can be provided in a range of settings, and in many countries, governments are in the process of shifting the emphasis away from long‐stay residential care to home care.^2^ Accordingly, a major issue in health and social care systems across many countries is how to keep dependent older people on the boundary of residential care living at home for longer, rather than be admitted to expensive long‐term care (LTC) facilities.^3^, ^4^ Not only is this what people want, but enabling older people to remain at home for longer reduces the potential cost of care for government.^5^ However, shifting the focus from LTC requires better information on older people in receipt of home care, which is in line with the World Health Organization (WHO)'s recommendation for improved measurement, monitoring, and understanding of health and ageing, including in relation to older people in need of LTC.^2^

The “Balance of Care” (BoC) approach, a systematic framework for exploring the potential costs and consequences of changing the mix of community and long‐stay residential services in a defined geographical area^6^ can be used to identify the types of dependent older people who could equally be cared for at home or in residential care.^7^, ^8^, ^9^ A large UK BoC study found that up to half of new care home entrants could be cared for in alternative settings.^9^ For each of these case‐types, nursing home care could be delayed by 3 to 12 months with sufficient community supports.

One of the enduring criticisms of government policy for dependent older people in Ireland is the imbalance in public spending between residential care and community‐based care. Currently, the government in Ireland is spending more than twice as much on residential care than on home care for older people; estimated at €962 million to support 23 334 people in LTC relative to a budget of €408 million for home care services to support approximately 49 000 people annually.^10^ Historically, even when public resources were more plentiful, investment in community‐based care has been relatively poor.^11^ Similar to UK BoC studies, O'Shea and Monaghan highlighted the potential of enhanced individualised supports for keeping people with dementia living in their own homes for longer in Ireland.^7^

Home support in Ireland is characterised by a complex mix of informal, public, and private home care, both in funding and provision.^12^, ^13^ As in many other countries, the bulk of home care in Ireland is informal, provided by family members/friends mostly in an unpaid capacity. Formal home care services are often used to supplement informal home care. Families can arrange private home care provision, funded from out of pocket payments. However, much formal home care is publicly (HSE) funded through taxation, which is arranged by the HSE but can be provided by different providers.

Publicly funded home support for older people in Ireland consists of assistance with domestic tasks, such as help with cleaning, cooking, and personal hygiene, as well as personal care services (bathing, dressing, grooming, etc). In 2017, the Health Service Executive provided approximately 49 000 older people with home support services, amounting to 16.3 million hours,^10^ suggesting that the average number of home support provision per recipient is just over 6 hours per week. Formal home support is allocated based on a care needs assessment by a health care professional and is currently not subject to a financial means assessment, although this may change as the Department of Health in Ireland is currently in the process of undertaking work to develop a new statutory formal home care scheme for older people.^14^

Keeping dependent people at home in the absence of sufficient community‐based resources is not an easy task.^15^, ^16^, ^17^ The types of cases that could be cared for in the community rather than in residential care tend, not surprisingly, to be those that are less complex.^4^, ^9^ Case types that are more likely to be viable for home support are those without a combination of high levels of physical dependency, cognitive impairment (CI), or challenging behaviours.^9^ All of the case types identified as being suitable for home care by Tucker et al had low levels of challenging behaviour.^4^ Women and younger people are also more likely to be viewed as suitable for home care.^9^ It may be difficult sometimes to target community supports at people who will derive most benefit from them, very often because of poor communication, particularly with people with dementia,^18^ and a failure to understand the importance of joint production in community‐based care. Even for people with a dedicated family carer, there may come a point where residential care is the most appropriate placement.^8^ However, making decisions about the most appropriate care setting or the optimal time for transition to long‐stay residential care is not straightforward. Saks et al have highlighted the many factors that need to be considered including preferences, quality of life of the person with dementia and family carers, quality of care, safety, service availability, costs, and cultural traditions and norms.^19^ In addition, the authors found that there is considerable variation among health professionals across several EU countries regarding the most appropriate setting in which to care for a person with dementia on the margins of home and residential care. Residential care, however, was seen as most appropriate for people with dementia with advanced CI, high levels of physical dependency, high behavioural problems, high levels of caregiver burden, and where caregiver has a positive attitude towards placement in residential care.^19^

Predicting the factors that influence admission to LTC or mortality among community‐based dependent older people is an important part of the resource allocation process, because it can help focus policy attention on the key factors that can prolong living at home and extend life in the community. There is evidence that reduced activities of daily living (ADL) activity is associated with functional deterioration, LTC, and mortality among older people.^20^, ^21^ People with higher levels of physical dependency and limitation may need to be admitted to LTC facilities because of their increased care burden and have a higher risk of death when incapacity becomes too severe.^20^ In a systematic review and meta‐analysis, Cepoiu‐Martin et al found that increased age, functional impairment, behavioural, and psychological problems and being in the severe stages of dementia were significant predictors of LTC placement for people with dementia.^22^

The older population in receipt of home support represent a vulnerable subgroup, characterised by a high prevalence of “cognitive dysfunction” (46%) and frailty (41%).^23^, ^24^ There have been no studies done on the identification of factors that influence admission to long‐stay care in Ireland, or on predictors of mortality among community‐dwelling older dependent adults. The aim of this paper is to identify factors that predict admission to LTC and mortality during a 1‐year period, based on a large cohort of community‐dwelling older adults receiving formal home support. Given that “ageing in place” is a dominant framework for care and support of older people, in many countries across the world, this study has international relevance.

## 2 METHODS

### 2.1 Study design

An audit was conducted of all community‐dwelling older adults receiving home support during 2017 in the Dublin North Central Health Service Executive administrative area.^10^ This area serves a population of 19 389 adults aged 65 years or older. Participants were identified based on the following inclusion criteria: adults aged 65 years and over, living at home in the defined community area, and receiving formal publicly funded home support during the audit period (January 2017‐December 2017). The data for this this study were extracted from the Common Summary Assessment Report (CSAR), which is a mandatory form completed by a community nurse or other health professional as part of the home support application and review process. Data from the CSAR forms are routinely collected administrative data and, while administrative data have its pitfalls, these data make it feasible and possible to address questions relating to health and social care services for older people that would be impractical or impossible to study using conventional research methods. 25 Written informed consent was obtained from all participants to complete the CSAR assessments. All data were anonymised before use. Analysis and evaluation of data were approved by the HSE, Community Health Organisation Area 9, Dublin, and by the Research Ethics Committee in Dublin City University, Dublin (DCUREC/2018_088).

### 2.2 Study variables

Sociodemographic data included age, gender, and personal circumstances (eg, marital status and living alone). Physical dependency level was assessed using the Barthel index (BI), a simple index based on the scoring of 10 items. Scores range from 0 to 20, with a lower score indicating a higher level of dependency. Its main aim is to establish the degree to which a person is independent from needing any help, physical or verbal, with these items however minor and for whatever reason. From the overall score, an individual is categorised as maximum dependency (0‐5), high dependency (6‐10), medium dependency (11‐15), low dependency (16‐19), and independent (20).^26^ Assistance with mobility and feeding (two of 10 items on the BI), falls risk (documented on CSAR form), and assistance with meal preparation (documented on care plan) were recorded as binary variables. Medications are listed on the CSAR form and polypharmacy, classified as use of five or more medications daily, was considered an indication of comorbidity. “Dementia” and “suspected CI” were based on information on cognitive status recorded on the CSAR form. The method used for the purposes of this audit has been previously described^24^ and is as follows. Service users were categorised as having (a) “dementia” if a diagnosis of dementia or cognitive decline with impact on independent living, was documented on the CSAR form by a health professional (geriatrician, public health nurse, general practitioner, occupational therapist) or (b) “suspected CI” where a validated cognitive screening tool was applied, and the documented score was indicative of mild CI. Service users with an absence of recorded evidence of a dementia diagnosis and those with a screening test score indicative of “non‐CI” in the absence of other dementia evidence, were categorised as “CI not suspected.” Collectively, people categorised as “dementia” and “suspected CI” were termed “cognitive dysfunction.” Ability to communicate is recorded in the CSAR form according to five categories: no problems, retains most information and can indicate needs verbally, difficulty speaking but retains information and indicates needs non‐verbally, can speak but cannot indicate needs or retain information, and finally, no effective means of communication. For the purposes of analysis in this study and as used in our previous study,^23^ these five categories were collapsed into two: no communication difficulties, which included those recorded as having “no problems” and communication difficulties, which included all others. Information available on home support came from the administrative dataset and included: duration of home support in months (from initial commencement of home supports), intensity of supports (measured by hours of care per week), and referral source (community or hospital). For each participant, the figure used to represent intensity of home support was the most recently recorded information available at the end of 2017 and for those for whom home supports had ceased, the figure used was that which was most recently recorded prior to the cessation of home support. When a person was admitted to LTC (nursing home care/institution) or died during the 12‐month audit period, this was recorded on the dataset, ie, predefined outcomes of LTC (admission to nursing home or residential care) and death comprise the dependent variables in this paper.

### 2.3 Statistical analysis

Descriptive statistics are presented as means and SD or counts and proportions for sociodemographic, health, and home support service characteristics. Differences between the groups were examined by independent student *t* tests, *χ* ^2^, or ANOVA, as appropriate. Normality was assessed visually using histogram matrix plots and ladder tables to identify appropriate transformations; Monte Carlo simulations were used to identify outliers.

Multiple logistic regression analysis was used to examine the factors associated with admission to LTC and mortality. The results are presented as odds ratios (OR) with 95% confidence intervals.^27^ Tests for linear relationships were performed by the inclusion of continuous predictor variables to the model and are reported as OR for a single unit change with 95% CI. In adjusted models, covariates were selected based on previous research findings, inferential statistics, and following stepwise regression to test the contribution of each predictor (excluded *Pr*. > 0.25). Unadjusted logistic regression results are also reported. All analyses were performed in STATA V14.0 (StataCorp LP).

## 3 RESULTS

### 3.1 Characteristics of community‐dwelling older adults receiving home support

A total of 1597 urban, community‐dwelling older adults, (≥65 years), in receipt of state‐funded home support were identified in this audit during the 12‐month period (January‐December 2017). The group was predominantly female (64%), widowed/single (65%), and over 80 years (68%), with a mean age of 83 years, as detailed in Table 1. Over half lived alone (55%) and were referred for home support assessment following hospitalisation (53%).

**Table 1:** Characteristics of older adults receiving home support, overall, and by gender (n = 1597)

	Overall	Female	Male	P value
Gender, n (%)	‐	1016 (63.6)	581 (36.4)	<.001*
Age in years, mean (SD)	83.59 (7.1)	84.5 (7.2)	83.0 (7.0)	<.001*
Age group, n (%)				
65‐69 y	73 (4.6)	44 (4.3)	29 (4.9)	.543
70‐74 y	132 (8.3)	77 (7.6)	55 (9.5)	.188
75‐79 y	240 (15.0)	136 (13.4)	104 (17.9)	.015*
80‐84 y	380 (23.8)	233 (2.9)	147 (25.3)	.285
85‐89 y	471 (29.5)	314 (30.9)	157 (27.0)	.102
90‐94 y	230 (14.4)	159 (15.7)	71 (12.2)	.060
≥95 y	71 (4.5)	53 (5.2)	18 (3.1)	.048*
Personal circumstances, n (%)				
Lives alone	864 (55.4)	600 (60.5)	264 (46.4)	<.001*
Widowed	680 (44.2)	509 (52.3)	171 (30.4)	<.001*
Married	477 (31.0)	226 (23.2)	251 (44.6)	<.001*
Single	313 (20.4)	209 (21.5)	104 (18.5)	.161
Divorced/separated	67 (4.4)	30 (3.1)	37 (6.6)	.001*
Home support characteristics				
Duration of home support (months)^a^	16 (20)	16 (20)	14 (20)	.054
Duration of home support (months)^b^	19.9 (18.3)	20.3 (18.0)	19.2 (18.7)	.255
Weekly care hours, mean (SD)	11.1 (7.2)	11.1 (7.2)	11.2 (7.2)	.8892
eHealth‐fold telecare, n (%)	140 (8.8)	92 (9.1)	48 (8.3)	.590
Referral source, n (%)				
Hospital	848 (53.1)	522 (51.4)	326 (56.1)	.068
Community	749 (46.9)	494 (48.6)	255 (43.9)	.068
ADL status				
Barthel index score^c^, mean (SD)	13.1 (3.9)	13.1 (3.9)	13.1 (3.9)	.9152
Level of dependency (BI)^d^, n (%)				
Independent	59 (3.7)	34 (3.4)	25 (4.3)	.33
Low	366 (24.3)	238 (23.4)	128 (22.0)	.524
Medium	726 (48.1)	464 (45.7)	262 (45.1)	.824
High	295 (19.6)	180 (17.7)	115 (19.8)	.304
Maximum	62 (4.1)	42 (4.1)	20 (3.4)	.491
“Cognitive dysfunction,” n (%)				
“Dementia”	277 (17.4)	178 (17.5)	99 (17.0)	.397
“Suspected cognitive impairment”	415 (25.9)	282 (27.8)	133 (22.9)	.022*
“Cognitive dysfunction”^e^	692 (43.3)	460 (45.3)	232 (39.9)	.04*
Polypharmacy^f^	1078 (67.5)	683 (67.2)	395 (67.9)	.754

a Median and IQR.

b Mean, (SD).

c Barthel Index score: Sum of scores from 10 items. Overall score ranges from 1 to 20 with lower scores indicating higher dependency.

d Level of dependency: based on categories used in Barthel Index—independent (score of 20), low dependency (score of 16‐19), medium dependency (score of 11‐15), high dependency (score of 6‐10), maximum dependency (score of 0‐5).

e Combined prevalence of “dementia” or “suspected cognitive impairment.”

f Polypharmacy: ≥5 medications reported.

* *p* < 0.05.

The majority were dependent in ADLs, with 72% categorised to be of medium‐maximum dependency according to the BI, reflecting the home care assessment process, which is largely based on physical dependency. Polypharmacy was high (68%), and over half of older people were identified as being at risk of falls (53%). The prevalence of either “dementia” or “suspected CI” (collectively termed “cognitive dysfunction” in this paper) was 43% in this population and was significantly higher in females than for males.

Home support intensity, as measured by allocated care hours, was on average 11 hours per week. Overall, 655 756 hours of state‐funded home support were delivered during the audit period. Median time since initiation of home support was 16 months. A small number of home support recipients concomitantly received eHealth telecare services (9%). During the 12 months, home support was discontinued for 19% (n = 304) of users. The predefined endpoints, namely, admission to LTC and mortality were 8% (n = 122) and 9% (n = 148), respectively. A small number of home support services were discontinued for unknown reasons (n = 21) or because of an acute hospital admission (n = 7).

### 3.2 LTC predictors

The subgroup of home support recipients admitted to LTC were characterised by a significantly (*P* < .05) higher prevalence of falls risk (71%), “cognitive dysfunction” (61%), support with feeding (42%), and communication difficulties (21%). They were also older and more dependant in terms of ADLs than people “ageing in place” at home, as detailed in Table 2. In the adjusted multivariate logistic regression model, “cognitive dysfunction” (ie, “dementia” or “suspected CI”) significantly predicted transition to LTC, but physical dependency (Barthel score) did not reach statistical significance (Table 3). The OR for “cognitive dysfunction” was 2.1. Home support intensity was significantly higher prior to admission to LTC, by an average of 3 hours per week, compared with those who continued to live at home and receive home support. Consistent with this difference, having higher weekly care hours was a significant predictor of LTC in the adjusted regression model. In line with this, people with “cognitive dysfunction” also received significantly more home support hours per week, accounted for by the need for support with meal preparation, which was significantly higher compared with others in this study (69% vs 58%, *P* < .001).

**Table 2:** Characteristics of community‐dwelling older adults admitted to long‐term care during a 12‐month period (n = 1415)

	Ageing in place (n = 1293)	Long‐term care (n = 122)	P value
Age in years, mean (SD)	83.1 (6.9)	84.5 (7.8)	.04*
Age group, n (%)			
65‐69 y	5.4 (4.2)	7 (5.7)	.417
70‐74 y	105 (8.1)	9 (7.4)	.773
75‐79 y	202 (15.6)	14 (11.5)	.223
80‐84 y	314 (24.3)	27 (22.1)	.595
85‐89 y	397 (30.7)	28 (22.9)	.074
90‐94 y	178 (13.8)	28 (22.9)	.006**
≥95 y	43 (3.3)	9 (7.4)	.023*
Service profile			
Weekly care hours,^a^ mean (SD)	10.8 (7.2)	13.5 (6.6)	<.001***
Dependency status			
Barthel index Score,^b^ mean (SD)	13.4 (3.8)	11.9 (3.9)	<.001***
Mobility assistance, n (%)	788 (60.9)	87 (71.3)	.02*
Feeding difficulties, n (%)	391 (30.2)	51 (41.8)	.009**
Communication difficulties, n (%)	166 (12.8)	26 (21.3)	.009**
Health‐related characteristics, n (%)			
“Cognitive dysfunction”	548 (42.4)	74 (60.7%)	<.001***
Falls risk	670 (51.8)	76 (71.3)	.02*

a Intensity of formal home support.

b Barthel Index Score: range from 1 to 20 with lower scores indicating dependency.

* *p* < 0.05.

** *p* < 0.01.

*** *p* < 0.001.

**Table 3:** Multivariable logistic regression model for factors associated with long‐term care admission, including all listed characteristics as potential influencing factors (n = 1415)

	Bivariate OR (95% CI)			Multivariate OR (95% CI)		
	OR	95% CI	P	OR	95% CI	P
Weekly care hours	1.04	1.02‐1.07	<.001	1.05	1.01‐1.06	.003*
Care plan: Feeding assistance	1.65	1.13‐2.42	.01	1.31	0.84‐2.04	.228
Falls risk^a^	1.54	1.05‐2.25	.03	1.31	0.87‐1.97	.199
“Cognitive dysfunction”^b^	2.09	1.44‐3.06	.001	2.10	1.41‐3.14	<.001**
Barthel index score^c^	0.92	0.87‐0.96	<.001	0.95	0.89‐0.99	.05

*Note*. OR: Odds Ratio, as derived from logistic regression model with long‐term care as the dependent variable.

a Health care professional documentation of person being at falls risk.

b This includes two groups of persons. The first group are those for whom the health care professional has documented an established diagnosis of dementia in the CSAR. The second group are those without a documentation of dementia diagnosis by the health professional, but where a cognitive test score was recorded in the CSAR, and this score fell outside a specified cut‐off point and for the purposes of this study were designated as having “suspected cognitive impairment.”

c Continuous predictor variable with a lower score indicating increased dependency on ADL's.

* *p* < 0.05.

** *p* < 0.01.

### 3.3 Mortality predictors

The subgroup of older adults who died during the 1‐year period were characterised by advanced age (>95 years), higher physical dependency, need for assistance with mobility, and need for assistance with meal preparation, as detailed in Table 4. Not surprisingly, the mortality group was receiving significantly more formal home support hours prior to death than those who remained ageing in place. The adjusted multivariate logistic regression model showed that age of ≥95 years [OR 4.45 (2.48, 7.98), *P* < .001] and higher dependency as indicated by a lower Barthel score [OR 0.92 (0.88, 0.97), *P* < 0.001] were significantly associated with mortality among home support recipients, as detailed in Table 5. “Cognitive dysfunction” was significantly associated with mortality, in contrast to the findings for admission to LTC.

**Table 4:** Characteristics of community‐dwelling older adults receiving home support who reached end of life over the course of 1 year compared with those who remained at home (n = 1441)

	Ageing in place (n = 1293)	End of life (n = 148)	P value
Age in years, mean (SD)	83.1 ± 6.9	84.5 ± 8.9	.09
Age group, n (%)			
65‐69 y	54 (4.2)	9 (6.1)	.283
70‐74 y	105 (8.1)	16 (10.8)	.264
75‐79 y	202 (15.6)	17 (11.5)	.184
80‐84 y	314 (24.3)	28 (18.9)	.146
85‐89 y	397 (30.7)	40 (27.0)	.357
90‐94 y	178 (13.8)	19 (12.8)	.755
≥95 y	43 (3.3)	19 (12.8)	<.001*
Service profile			
Referral‐acute hospital	418 (32.3)	58 (39.2)	.09
Subacute hospital	252 (19.5)	32 (21.6)	.537
Community	623 (48.2)	58 (39.2)	.04*
Weekly care hours,^a^ mean (SD)	10.8 (7.2)	12.6 (6.9)	<.001*
Dependency status			
Barthel index score.^b^ mean (SD)	13.4 (3.8)	11.9 (4.5)	<.001*
Meal preparation assistance, n (%)	789 (61.0)	105 (70.9)	.02*
Mobility assistance, n (%)	788 (60.9)	111 (75.5)	.004*

a Intensity of formal home support.

b Barthel Index Score: range from 1 to 20 with lower scores indicating dependency.

* *p* < 0.05.

**Table 5:** Multivariable logistic regression model for determinants' of end of life, including all listed characteristics as potential factors (n = 1441)

	Bivariate OR (95% CI)			Multivariate OR (95% CI)		
	OR	95% CI	P	OR	95% CI	P
Home support (h)^a^	1.03	1.01‐1.05	.001	1.02	0.99‐1.04	.138
Hospital referral^b^	1.44	1.02‐2.04	.04	1.37	0.95‐1.98	.089
Age 95+	2.65	1.09‐6.45	.031	4.45	2.48‐7.98	<.001*
Care plan: Meal preparation	1.56	1.08‐2.26	.02	1.44	0.98‐2.13	.063
Barthel index score^c^	0.92	0.88‐0.96	<.001	0.92	0.88‐0.97	<.001*

*Note*. OR; Odds Ratio, as derived from logistic regression model with End of Life as the dependent variable.

a Weekly state‐funded formal Home Support hours.

b Includes acute and sub‐acute referral sources.

c Continuous predictor variable with a lower score indicating increased dependency on ADL's.

* *p* < 0.05.

## 4 DISCUSSION

The aim of this study was to assess and compare predictors of admission with LTC and mortality for dependent older people currently living at home and in receipt of state‐funded home support. Our findings showed that “cognition dysfunction” was an important predictor of LTC while physical dependency and advanced age were associated with mortality in this population. This is an important cohort of people whose profile and needs have been overlooked in public policy in Ireland. The only statutory scheme for dependent older people in Ireland is the Nursing Home Support Scheme (NHSS), which is focused on admission to LTC and provides an element of certainty and protection in the residential long‐term sector compared with home care provision, where there is no statutory coverage. It is no surprise, therefore, that the Irish Government is now taking steps to develop a statutory response to deficiencies in community‐based provision through a new Home Care funding scheme drawing on experiences of other countries.^14^ However, moving from the current fragmented system of community‐based care to a rights‐based model will take time and will be expensive to implement.^27^ This paper provides some insight into where future investment should be concentrated, particularly if the goal is to slow‐down or postpone admissions into LTC and the reduction of avoidable mortality.

The results suggest, in keeping with the international literature,^22^, ^28^, ^29^ that a greater focus on “cognitive dysfunction” would likely yield dividends in postponing admission into LTC for some older people. In the present study, the prevalence of “cognitive dysfunction” was 43.1% consistent with our previous findings among home support recipients.^24^ While there is generally a dearth of research on the prevalence of “cognitive dysfunction” among older people in receipt of home support, evidence from other countries (eg, Norway and Canada) suggests that people with “cognitive dysfunction” are relatively high users of home care services^30^, ^31^ In the present study, “cognitive dysfunction” was found to be significantly associated with LTC. There is a growing realisation of the importance of providing a rich and varied individualised set of responses to dependent older people with cognitive dysfunction,^11^ many of which may be feasible to embed within home support. One of the few areas that has been positively evaluated in relation to cognitive dysfunction is cognitive stimulation therapy (CST) for people with dementia, a nonpharmacological intervention aimed to stimulate people's cognition in a positive learning and social environment. Systematic reviews have found cognitive stimulation for people with mild to moderate dementia has robust evidence,^32^, ^33^, ^34^ improving communication, social interaction, and quality of life for people with dementia. The implementation of such approaches could yield significant gains in terms of preventing unnecessary admissions for some people into LTC.

The present study also indicates that admission to LTC is significantly associated with intensity of home care hours. It may be that the number of hours available was below the critical level needed to support people with significant “cognitive dysfunction” to remain living at home. Given the importance of “cognitive dysfunction” among participants, one would hope that provision would focus disproportionately on addressing cognitive needs. Unfortunately, despite recent progress, Ireland is not yet characterised by a responsive personalised social care system.^27^ Hence, it is likely that the care hours provided were generic in nature, concentrating as much, if not more, on assistance with practical tasks and personal care than on addressing the cognitive health and well‐being of recipients.

The importance of physical dependency for mortality is supported by the literature^20^ and raises interesting questions about the constituent elements of home support in Ireland. The narrowness of social care provision in the country has been well documented.^35^ Of particular relevance, in view of the mortality finding is the absence of a co‐ordinated, holistic, activity programme for dependent older people living at home, focusing on movement, flexibility, mobility, and exercise. The timeframe for home support in the present study was a median of 16 months and in total represented in excess of 656 000 formal home support contacts, suggesting ample opportunity for personalised multicomponent interventions to impact on physical dependency and its consequences.^36^, ^37^ Generally, those receiving home support represent a heterogeneous subgroup of community‐dwelling older people with complex health and social care needs.^23^, ^24^, ^38^ Models of home support should, therefore, reflect this complexity, and address cognitive and physical dependency as highlighted in this study, including a broader range of personalised supports for social isolation, mental health, cultural, and personal aspects.^37^, ^39^, ^40^, ^41^, ^42^ This approach has been recommended by the WHO.^2^

A strength of this study is the large sample size. The administrative data were routinely collected as part of home support application and review processes and are an important source of information about health and social care delivery. However, it is also recognised as having inherent limitations.^43^ There are a number of important limitations to this paper. A common criticism of administrative data relates to the accuracy with which diagnoses are recorded. This presented a major challenge for the authors of this study, who developed an algorithm to identity people with dementia. Although the approach used to estimate prevalence of “cognitive dysfunction” has its flaws, in the absence of higher quality data, it is a judicious attempt to identify people with “dementia” and “suspected CI” using home supports. Another key limitation is that important variables of interest were not available; information on educational and economic status of care recipients was lacking, as was more detailed information on health including level of dementia and presence of neuropsychiatric symptoms, and cognitive and social support. A further key limitation of the CSAR, in the context of this paper, is the absence of information on family carers. This is a major deficiency given the important role played by carers in supporting dependent older people to remain in their own home for as long as possible.^11^, ^44^, ^45^ There is also evidence that higher caregiver burden is an important predictor of admission to LTC.^29^, ^46^, ^47^ The absence of clear information on diagnosis of dementia, level of dementia, presence of neuropsychiatric symptoms, and caregiver burden is not only a limitation of this study but it also has important repercussions for the planning and provision of home supports, as without a comprehensive assessment, it is hard to see how home supports can be adequately tailored to the individual needs of people with dementia and their family carers. As Molony et al have argued, “the quality of dementia care rendered to individuals and families is contingent on the quality of the assessment and care planning, and the degree to which these are person‐centred.”^48^ Despite its limitations, these data are the best evidence on home supports for older people in Ireland available at this time. The strength of these findings is that it is based on “real world” data on a large population and will be helpful to policymakers when making decisions between funding options for home support and residential care. Such decisions are faced by policymakers in many countries around the world.

## 5 CONCLUSION

“Cognitive dysfunction” and higher levels of community care provision are important predictors of admission to LTC among dependent older people living at home in Ireland. Advanced age and physical dependency are key determinants of mortality in this cohort. The findings support the evidence and case for personalised multimodal home support models. This is a priority for older adults with dementia and CI in order to support them to live well at home for longer.

## CONFLICT OF INTEREST

The authors have no conflicts of interest to disclose.
